# Determinants for cardiovascular disease health check questionnaire: A validation study

**DOI:** 10.1371/journal.pone.0188259

**Published:** 2017-11-16

**Authors:** Ai Theng Cheong, Karuthan Chinna, Ee Ming Khoo, Su May Liew

**Affiliations:** 1 Department of Family Medicine, Faculty of Medicine and Health Sciences, Universiti Putra Malaysia, Serdang, Selangor, Malaysia; 2 Department of Social and Preventive Medicine, Faculty of Medicine, University of Malaya, Kuala Lumpur, Malaysia; 3 Department of Primary Care Medicine, University of Malaya Primary Care Research Group (UMPCRG), Faculty of Medicine, University of Malaya, Kuala Lumpur, Malaysia; George Institute for Global Health; Sydney Medical School, University of Sydney, AUSTRALIA

## Abstract

**Background:**

To improve individuals’ participation in cardiovascular disease (CVD) screening, it is necessary to understand factors that influence their intention to undergo health checks. This study aimed to develop and validate an instrument that assess determinants that influence individuals’ intention to undergo CVD health checks.

**Methods:**

The concepts and items were developed based on findings from our prior exploratory qualitative study on factors influencing individuals’ intention to undergo CVD health checks. Content validity of the questionnaire was assessed by a panel of six experts and the item-level content validity index (I-CVI) was determined. After pretesting the questionnaire was pilot tested to check reliability of the items. Exploratory factor analysis was used to test for dimensionality using a sample of 240 participants.

**Results:**

The finalized questionnaire consists of 36 items, covering nine concepts. The I-CVI for all items was satisfactory with values ranging from 0.83 to 1.00. The exploratory factor analysis showed that the number of factors extracted was consistent with the theoretical concepts. Correlations values between items ranged from 0.30 to 0.85 and all the factor loadings were more than 0.40, indicating satisfactory structural validity. All concepts showed good internal consistency, Cronbach’s alpha values ranged 0.66–0.85.

**Conclusions:**

The determinants for CVD health check questionnaire has good content and structural validity, and its reliability was established. It can be used to assess determinants influencing individuals’ intention to undergo CVD health checks.

## Introduction

Cardiovascular disease (CVD) is a major cause of death and health burden globally [1]. Low- and middle-income countries are the most affected [2, 3] where the prevalence and lack of awareness of cardiovascular (CV) risk factors remain high [4]. Thus, health checks are important and necessary for early detection of individuals at high CV risk to allow for timely intervention. However, the uptake rate of CVD health checks remains low with values ranging from 20% to 40% [5, 6].

To improve participation rates of health checks for prevention of CVD, it is crucial to understand factors that influence individuals’ intention to go for such checks. We addressed this by first exploring factors that influenced individuals’ decision-making to undergo health checks for CVD prevention in a previous qualitative study [7]. Following this, a questionnaire was developed to identify the factors that influenced individuals’ intention to undergo health checks for CVD prevention using a quantitative study on a population.

For an instrument to measure determinants for individuals’ intention to undergo CVD health checks, it needs to assess all factors (concepts) that are being measured [8]. A number of questionnaires have been used in previous studies to examine factors influencing individuals’ intention to participate in health checks using the health belief model [9–12] or the theory of planned behaviour [13,14]. However, the items used were not grounded to local context and the concepts measured did not include all determinants found in our qualitative study [7]. Thus, we aimed to develop and validate a questionnaire that assessed all determinants that could influence individuals’ intention to undergo CVD health-checks.

## Methods

The development of this questionnaire involved two steps. First the concepts and items for the intended measures were developed. Next the internal validity of the questionnaire was assessed.

### Development of concepts and related items

The concepts that represent the determinants of individuals’ intention to undergo CVD health checks were developed based on findings from our previous qualitative study [7]. The qualitative study used a grounded theory approach to develop a conceptual framework to explain individuals’ decision-making process to undergo CVD health checks, taking into account local and cultural context [7]. Participants from three main ethnic groups in Malaysia, Malay, Chinese and Indian were recruited [7]. Nine concepts were identified for the study conceptual framework:

a Individuals’ readiness to know the results of CVD health checks
b Individuals’ readiness to handle outcomes following CVD health checks
c Individuals’ beliefs that the course of CVD can be changed for the better
d Perceptions of self being at risk of CVD
e Perception of benefits of CVD health checks
f Perception of drawbacks of CVD health checks
g Preferred method for CVD prevention
h External barriers
i Influence by significant others

**Fig 1:**
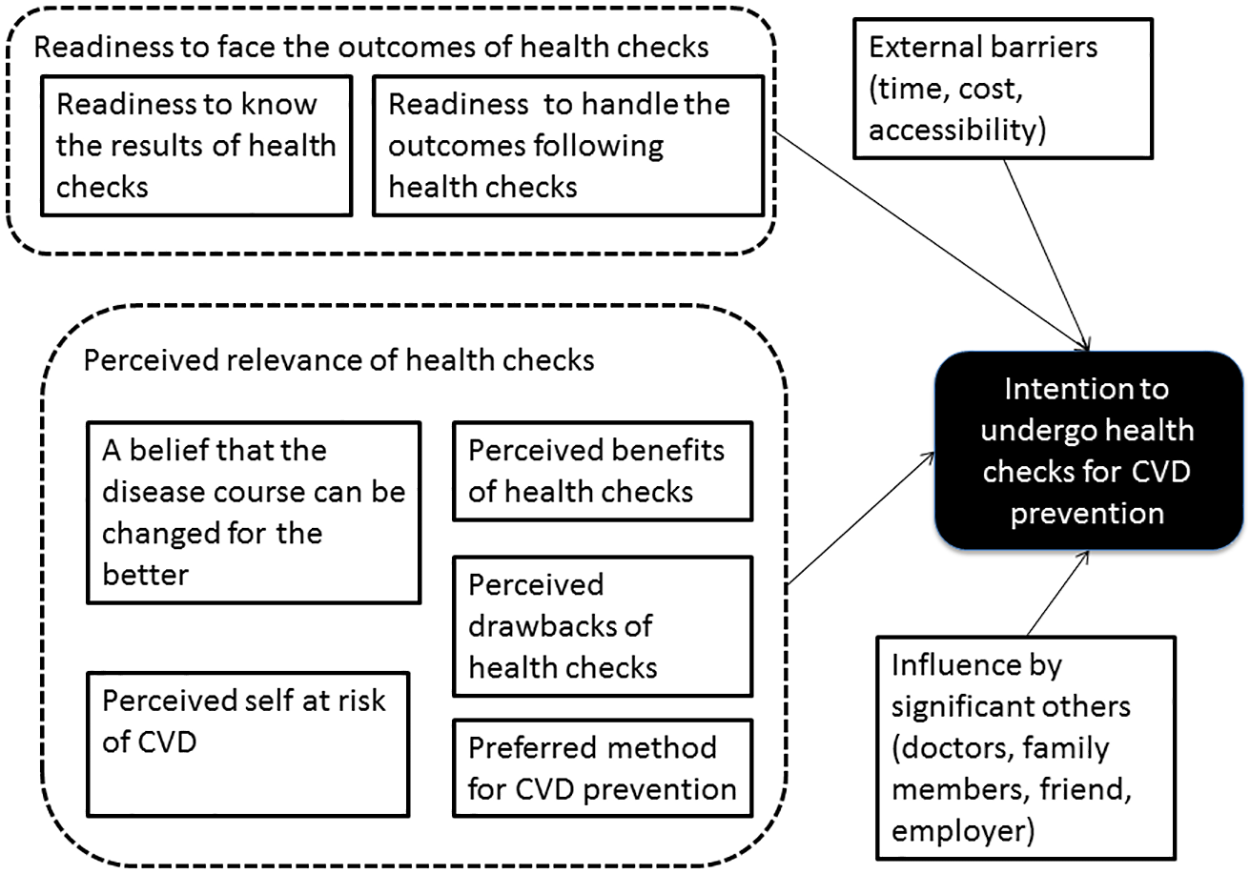
Conceptual framework: Factors that influenced individuals’ intention to undergo health checks for CVD prevention.

The first two concepts (a & b) measured individuals’ readiness to face outcomes of CVD health checks. The next five concepts (c, d, e, f & g) measured individuals’ perceived relevance of health checks for CVD prevention. The next concept (h) measured external barriers towards CVD health checks while the last concept (i) measured influence by significant others (refer Fig 1). Table 1 describes operational definitions of these concepts.

**Table 1:** Operational definition of concepts that influence individuals’ intention to undergo health checks.

Concepts	Operational definition
Readiness to know the results of CVD health checks	One’s preparedness to receive results of CVD health checks.
Readiness to handle outcome following CVD health checks	One’s preparedness to deal with management arising from results of CVD health checks.
A belief that the course of CVD can be changed for the better	A belief that CVD is preventable and treatable.
Perception of self being at risk of CVD	Perceived self being vulnerable to CVD.
Perceived benefits of CVD health checks	Perceived gains from undergoing health checks.
Perceived drawbacks of CVD health checks	Perceived disadvantages in undergoing health checks.
Preferred method for CVD prevention	Preferred medical measures such as health checks and medical treatment or healthy lifestyle for CVD prevention.
External barriers	Barriers in terms of time, cost and accessibility.
Influence by significant others	Significant others are people who have the influence to encourage or discourage one’s intention to undergo health checks.

Items in each concept were statements derived from themes that emerged and wording expressed by participants in the earlier qualitative study [7]. A Likert scale of 1 to 5 was used to indicate participant’s level of agreement with each item; 1 indicated “strongly disagree” and 5 “strongly agree”.

### Internal validation of the instrument

**Fig 2:**
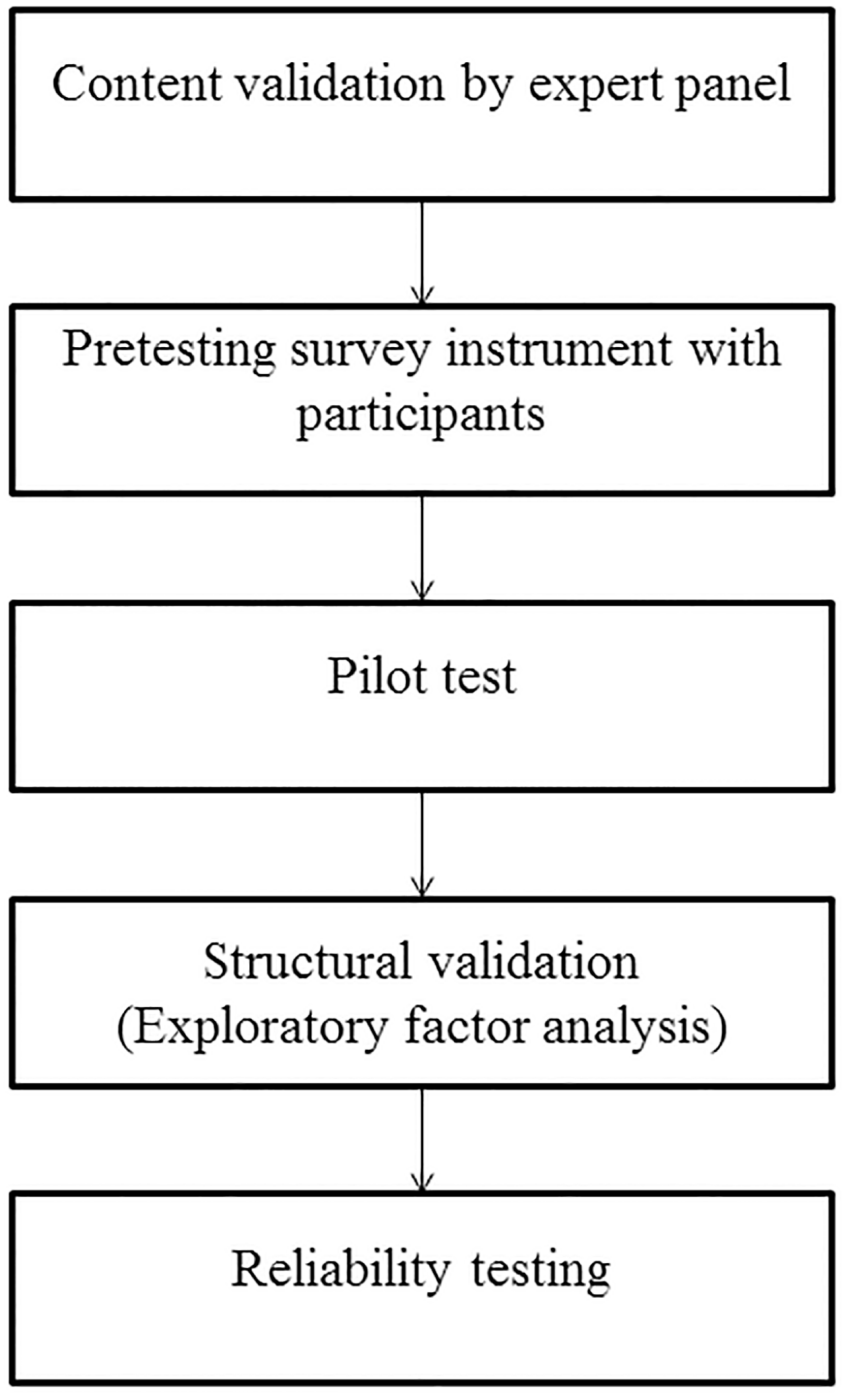
Internal validation process for questionnaire development.

The aim of internal validation is to establish if an instrument is valid and reliable to accurately measure what it is supposed to measure [8,15]. In this study, the process of validation consisted of content validation by an expert panel, pre and pilot testing of the questionnaire, assessment of correlation between items, structural validation using exploratory factor analysis and reliability analysis, both internal consistency and test-retest reliability (refer to Fig 2).

#### Content validation

The aim of content validation is to examine whether the content of the developed instrument is relevant, important and adequately representing the underlying concept [8,15–17]. In this study content validation was performed by an expert panel that comprised of six content experts: four academic family physicians, one family physician from a public primary care clinic and a psychologist.

The first draft of the questionnaire consisted of 41 items that addressed the underlying concepts. The draft was emailed to members of the panel for their ratings and comments. Each member rated each item according to its relevance to the underlying concept using a 4-point scale: not relevant (score 1) to highly relevant (score 4). For validation purpose, scores of 3 or 4 were taken as indicative of relevance [17]. An item–level content validity index (I-CVI) was the proportion of experts in agreement with relevance of the contents. It was computed as follows:

$$I-CVI\;=\;\frac{Numbers\;of\;experts\;giving\;a\;rating\;of\;either\;3\;or\;4}{Total\;number\;of\;experts}$$

Items with I-CVI of 0.78 or higher were considered to have good content validity [17,18]. Feedback and comments from the expert panel were used to further refine the items for better clarity and to provide representativeness of the concepts. In cases where items were modified, added or removed, the rating and review process was repeated.

#### Pretesting of questionnaire

After content validation process, a face-to-face interview was carried out with participants to pretest the items. Pretesting of an instrument involved testing the questionnaire with participants to ensure it was easily understood and met the purpose of what it was intended to measure [19]. This process aimed to assess readability and clarity of the items in terms of consistency and appropriateness of interpretations from participants [19].

A total of six participants, a male and female each from three main ethnic groups in Malaysia i.e. Malay, Chinese and Indian, were recruited from the community to pretest the questionnaire. Participants from different ethnic groups were recruited to ensure the questionnaire was culturally sensitive and appropriate for use in local community with multi-ethnic background.

Six interview sessions were carried out. During each session, participants first completed the questionnaire on their own. Subsequently, the researcher (ATC) asked participants to report their thoughts on each item. Clarity of the items and ease of completing the questionnaire were assessed. Feedbacks from the participants were used to further improve readability and clarity of the questionnaire.

#### Pilot test

A pilot study was conducted among 40 participants at a hypermarket to assess the internal consistencies of the items in the constructs. In the analysis, the correlation values between the items in the respective constructs were checked. In cases where the highest correlation of items with other items in the construct was less than 0.30, the items were modified and a second pilot test was carried out.

#### Study setting and population

In this validation study participants were recruited from an urban hypermarket, a very large store selling groceries and household products. The hypermarket was selected purposively as it was surrounded by housing estates with mixed population of varied socioeconomic background. Participants were community dwelling adults who visited the hypermarket, were Malaysians, and aged 30 years or older. The 30 years or older age group was the group recommended for screening of CVD risk factors by the Malaysian Ministry of Health [20]. Those with known history of stroke or coronary heart disease or who could not understand the Malay language (national language of Malaysia) were excluded.

#### Structural validation (exploratory factor analysis)

Factor analysis is a statistical method used to check dimensionality among items [15]. For a good construct, the items must be adequately inter-correlated to be grouped under a single dimension or factor [8, 15]. In this study, factor analyses were performed on the items in each construct separately. The sample size for the exploratory factor analysis was estimated based on the rule of thumb of five participants for every item [21, 22]. Hence for 36 items in this questionnaire, a minimum sample size of 180 participants was needed.

Before factor analysis, the correlation among the items in the construct was checked. Ideally, the highest correlation of an item with at least one other item in the construct should be between 0.30 and 0.85. Values less than 0.30 indicate non convergence and values more than 0.85 indicate multicollineatity.

In extraction, the principle axis factoring method was used and rotation was performed using the promax oblique rotation method [15, 21]. Sampling adequacy was assessed using Kaiser-Meyer-Olkin (KMO) value, where a KMO value ≥ 0.60 was considered to be acceptable [15]. The Bartlett’s test of sphericity was used to test if the correlation between the items was sizable. In factor analysis, the factor loadings must be > 0.40 [15].

#### Reliability testing

Reliability analysis is an assessment of the degree of consistency between multiple items in a construct [15]. In this study, internal consistency of the items was assessed using Cronbach’s alpha, where a value of ≥0.60 is considered reliable and acceptable [8, 15].

The test-retest reliability assesses the stability and reliability of an instrument over time [8, 15].

In this study, both unweighted and weighted kappa (both linear and quadratic weights) were tested to obtain a range of possible results. A kappa coefficient of ≥0.60 is desirable [8, 23].

### Data analysis

All statistical analyses were performed using IBM SPSS Statistics software version 22, except for weighted kappa which was performed using a statistical calculator from the Statistical Computation Web Site [23]. Data were cleaned before analysis was conducted. The responses for the items that were worded oppositely were reverse coded. For example, a reverse coding was carried out for the item “I don’t want to think and know about CVD diseases at all”.

### Ethical issues

Participation in the study was voluntary. The respondents were explained on the purpose of this study and were assured that the information would be kept strictly confidential and used for the purpose of this study only. Consent was obtained from willing respondents before the questionnaire was administered.

For this study ethical approval was obtained from the Medical Ethics Committee, University of Malaya Medical Centre (20145–274).

## Results

### Content validation

The first draft had 41 items with seven underlying concepts and one question asking participant’s intention to undergo health checks. Following feedback from the expert panel, six items were removed, and seven items were added, 16 items were refined and rephrased. The six items were removed because they were seen as not reflecting the underlying concept (I-CVI<0.78) or had strong similarities with other items. New items were added to widen the extent of representativeness of the content for the measured concepts. The revised questionnaire with 42 items was then sent to the same expert panel for re-evaluation. The I-CVI values for all items in the revised questionnaire ranged from 0.83 to 1.00.

In the pre-test, generally, the participants found the questionnaire to be easy and readable. Only minor revisions were made substituting more commonly used terms to improve clarity of the items.

Based on the results from the first pilot test, the initial concept of “perceived benefits and drawbacks of health check” were reclassified into two concepts i.e. “perceived benefits of health check” and “perceived drawbacks of health check”; the initial concept of “readiness to face outcome of health check” was also reclassified into two concepts “readiness to know the results of health checks” and “readiness to handle outcome following health check”. Seven items were deleted based on poor correlation (r <0.30) with other items. Two new items were added, and this revised version was sent to expert panel for re-evaluation. The I-CVI was satisfactory with index ranging from 0.83 to 1.00. The second version of the questionnaire consisted of 37 items. It was pilot tested again on another 40 participants. One item was deleted due to poor correlation, and the final version had 36 items with nine underlying concepts. The number of concepts and items in the initial and revised version are shown in S1 Appendix. Table 2 presents the concepts and respective items. The questionnaire was written in Malay (refer to S2 Appendix) and the translated version is shown in Table 2.

**Table 2:** Concepts and their related items.

Concepts	Items
A belief that the course of CVD can be changed for the better	(A1) I believe CVD (for example heart disease, stroke, etc.) can be prevented
	(A2) I believe early treatment of CVD risk factors (for example high blood pressure, high cholesterol level, diabetes mellitus) can prevent CVD
	(A3) I believe CVD is treatable
	(A4) If CVD can be detected early, the treatment will be easier
Perception of self being at risk of CVD	(B1) I am at risk of CVD
	(B2) My current age puts me at risk of CVD
	(B3) My lifestyle puts me at risk of CVD
	(B4) Medical problems in my family members put me at risk of CVD
	(B5) My current health condition puts me at risk of CVD
Preferred method for CVD prevention	(C1) For CVD prevention, I think practicing a healthy lifestyle is sufficient (for example healthy diet, exercise, qi gong, etc.)
	(C2) For CVD prevention, I prefer to adopt a healthy lifestyle than to undergo CVD health checks
	(C3) For CVD prevention, I am more confident with practicing a healthy lifestyle than using medical treatment
Perceived benefit of CVD health checks	(DB1) I feel undergoing a CVD health check will give assurance for my health
	(DB2) We will not know our CVD health status if we do not undergo CVD health checks
	(DB3) CVD health checks can act as an indicator for CVD prevention
	(DB4) CVD health checks enable us to detect risk factors of heart disease/stroke early
Perceived drawbacks of CVD health checks	(DD1) A CVD healthcheck is a waste of time
	(DD2) A CVD health check is a waste of money
	(DD3) A CVD health check involves a troublesome procedure (e.g. the need to fast before blood tests)
	(DD4) A CVD health check which finds abnormal health results will give rise to problems (e.g. affect the chance of purchasing insurance or securing a job).
Readiness to know the result of CVD health checks	(RFR1) I am ready to face the results of the CVD health check
	(RFR2) I want to know my CVD health status
	(RFR3) I don’t want to think and know about CVD diseases at all*
Readiness to handle outcomes following CVD health checks	(RHO1)If the CVD health check results are abnormal, I am ready to take medication
	(RHO2)If the CVD health check results are abnormal, I am ready to adjust my lifestyle
	(RHO3)If the CVD health check results are abnormal, I am ready to bear the cost of subsequent treatment
	(RHO4)If the CVD health check results are abnormal, I am not ready to do anything*
External barriers	(F1) I will make an effort to allocate time to go for a CVD health check*
	(F2) The cost of doing CVD health checks is a burden for me
	(F3) The place for CVD health checks is far from my house/workplace
	(F4) I have a problem with transportation to go for CVD health checks
Influence by significant others	(G1) I will perform CVD health check if recommended to do so by doctors
	(G2) I will perform CVD health check if my family member advises me to do so
	(G3) I will perform CVD health check if my friend advises me to do so
	(G4) I will perform CVD health check if my employer requires me to do so
	(G5) I will perform CVD health check as people around me have already done so.

*reverse scoring was performed

### Factor analysis and internal consistency

For the final analysis, a total of 605 shoppers were approached and out of this 240 agreed to participate, giving a response rate of 39.7%. The 240 participants were recruited in a similar manner as in the pilot-test for factor analysis.

The median age of the participants was 45.5 years (IQR 17 years), mean age was 46.4 years (SD 11.1 years). The majority of participants were females (60%) and of Malay ethnicity (53.3%). Nearly all participants were aware of heart attack (97.8%) and stroke (98.7%). About one-half (55.0%) of the participants had regular health checks, at least once in two years. Table 3 shows the profiles of the participants.

**Table 3:** Profiles of participants for factor analysis.

Characteristics		Frequency	Percentage
Gender, n = 240	Male	96	40
	Female	144	60
Age group (years), n = 240	30–39	78	32.5
	40–49	72	30.0
	50–59	52	21.7
	≥60	38	15.8
Ethnicity, n = 240	Malay	128	53.3
	Chinese	88	36.7
	Indian	15	6.3
	Others*	9	3.8
Education level, n = 240	Primary	8	3.3
	Secondary	105	43.8
	Tertiary	127	52.9
Marital status, n = 240	Never married	20	8.3
	Widow/widower	6	2.5
	Separated	13	5.4
	Married	201	83.8
Working status, n = 239	No	73	30.5
	Yes	166	69.5
History of co-morbidities, n = 239	Diabetes	28	11.7
	Hypertension	51	21.3
	Hypercholesterolemia	43	18.0
	Overweight/obesity	58	24.3
	Smoking	25	10.5
Family history of CVD, n = 239	No	136	56.9
	Yes	103	43.1
Awareness of CVD, n = 239	Heart attack	234	97.9
	Stroke	236	98.7
Health check experience, n = 240	Having any form of health check experience	225	93.8
Regular health check experience, n = 238	At least once a year	100	42.0
	Once in two years	31	13.0

*dusun, sikh, iban, kadazan, melanau, bidayuh

Factor analyses were performed on the items in each of the nine constructs separately. For each construct, the highest correlation of each item with at least one other item was between 0.30 and 0.85, indicating that all items were correlated adequately (refer S3 Appendix). The Keiser-Meir-Olkin (KMO) values ranged from 0.58 to 0.79. According to Hair et al (2010), a KMO value of > 0.60 is acceptable [15]. The Bartlett test of sphericity was statistically significant for all constructs, indicating that there were sufficient correlation among the items to support the application of factor analysis. The total variance extracted for the constructs ranged from 35.1% to 59.4% and the Cronbach’s alpha values ranged from 0.66 to 0.85. The lowest factor loading value was 0.44.

In test-retest reliability analyses involving 88 participants, the weighted kappa values showed fair to substantial agreement (refer to S4 Appendix).

## Discussion

Overall, the internal validity of the questionnaire was found to be acceptable.

This questionnaire has certain similarities and differences from existing questionnaires in medical literature. The concepts measured in this questionnaire; perception of CVD risk, possibility of a change in CVD outcomes and perceived benefits of health checks, are akin to concepts in the Health Belief Model [24]. The pperceived benefit and drawbacks of health checks constructs were similar to concept of attitude towards behaviour in theory of planned behaviour [25]. Significant others such as doctors, friends, spouse or relatives could influence individuals’ intention for health check participation. These social influences resemble the perceived social norms in theory of planned behaviour, which could motivate or demotivate an individual’s behavioural intention. The environmental factors are the external resources, such as financial support from the workplace and availability of nearby facilities or outreach programmes for health checks. Self-efficacy is an individual’s ability to find time, money or transport to undergo health checks [25]. In contrast with other questionnaires, this questionnaire is more comprehensive as it included additional concepts such as readiness to handle outcome and to face results of CVD health checks, which were raised in literature [11, 26–32].

To the best of our knowledge, this is the first questionnaire developed locally that assessed individuals’ intention to undergo CVD health checks. All other questionnaires were developed and used in Western countries such as United Kingdom and Netherlands. However, details of validation process of those questionnaires are lacking. A comparison of the current questionnaire with other similar questionnaires is provided in Table 4 [11, 14, 33].

**Table 4:** Comparison of current questionnaire with other CVD health check questionnaire.

Characteristics of study	Current study	Norman et al, 1991[11]	Norman et al, 1996[14]	Petter et al, 2015[33]
Country	Malaysia	United Kingdom	United Kingdom	Netherlands
Theory/conceptual framework	conceptual framework from earlier phase of qualitative study	Health belief model and items from previous literature	Theory of planned behaviour	NA
Concepts	Readiness to know results of CVD health checks Readiness to handle outcomes following CVD health checks A belief that the course of CVD can be changed for the better Perceptions of self being at risk of CVD Perceived benefits of CVD health checks Perceived drawbacks of CVD health checks Preferred method for CVD prevention External barriers Influence by significant others	General health beliefs Perceived susceptibility Perceived severity Perceived benefits Perceived barriers	Attitude Subjective norm or perceived social pressure Perceived behavior control	Practical factors Personal beliefs about own health and lifestyle
Exploratory factor analysis	Yes	NA	NA	NA
Cronbach alpha	0.658 to 0.845	0.53 to 0.95	0.54 to 0.93	NA

NA: information not available

### Recommendations/implications

The questionnaire developed in this study has the potential to help assess an individual’s concern when deciding whether to go for CVD health checks. This information can be used to provide an appropriate advice based on his/her concerns to facilitate their decision-making process to undergo health checks. Tailored messages are perceived to be personally relevant and can increase one’s receptivity to the information presented [34–36]. For example, for opportunistic health check invitations, this questionnaire can be given to potential participants to explore their concerns and intentions about CVD health check, which in turn can be addressed during consultations. This can facilitate shared decision-making between the individual and the health care provider. The questionnaire can also be used by public using web or computer-based application. Based on the information provided by the respondents, tailored messages can be sent to them to facilitate decision-making for CVD health checks.

### Strength and limitations

The strength of this questionnaire lies in the fact that the factors and items were grounded on an earlier qualitative study that explored understanding of individuals’ intention to undergo health checks, rather than a hypothesized theory. In this validation study, 240 participants were recruited. The sample size was adequate to address the factor analysis and internal consistency (minimum sample 180 for 36 items). However, due to the limitation of convenience sampling, generalisability of results is cautioned. Nevertheless, it was tested to be valid for use in a multi ethnic society which widens its applicability. More validation studies should be performed in different populations or subgroups.

## Conclusion

The questionnaire “Determinants of individuals’ intention to undergo CVD health check” is found to be a valid instrument with good construct and internal consistency. It can be used to determine factors influencing individuals’ intention to undergo CVD health checks so that targeted approach can be taken to improve participation of health checks for prevention of CVD.

## Supporting information

S1 Appendix Comparison of concepts and number of items in the initial and revised version of the questionnaire used for factor analysis. (DOCX)

S2 Appendix The “determinants of intention to undergo CVD health checks” questionnaire. (DOCX)

S3 Appendix The mean, standard deviation of items and correlation matrix of each concept. (DOCX)

S4 Appendix Summary of test-retest reliability for all items in the questionnaire. (DOCX)
