# Supplementary material for: Determinants for cardiovascular disease health check questionnaire: A validation study
Source: PLoS One. 2017 Nov 16;12(11):e0188259. doi: 10.1371/journal.pone.0188259 (PMC5690630; doi:10.1371/journal.pone.0188259)
Supplement: S1 Appendix — (DOCX) [file pone.0188259.s001.docx]

**S1 Appendix: Comparison of concepts and number of items in the initial and revised version of the questionnaire used for factor analysis**

|  | **Initial version** | | **Revised version for factor analysis** | |
| --- | --- | --- | --- | --- |
|  | **Concepts** | **no. of items** | **Concepts** | **no. of items** |
|  | Believe that the disease course can be changed for better outcomes | 6 | Believe that the disease course can be changed for better outcomes | 4 |
|  | Perceived self at risk of CVD | 4 | Perceived self at risk of CVD | 5 |
|  | Preferred method for CVD prevention | 5 | Preferred method for CVD prevention | 3 |
|  | Perceive benefit and drawbacks of health checks | 8 | Perceived benefit of health checks | 4 |
|  |  |  | Perceived drawback of health checks | 4 |
|  | Readiness to face the outcome of the health checks | 6 | Readiness to know the result of health checks | 3 |
|  |  |  | Readiness to handle the outcomes following health checks | 4 |
|  | External barriers | 8 | External barriers | 4 |
|  | Influence by significant others | 4 | Influence by significant others | 5 |
| Total concepts/ items | 7 | 41 | 9 | 36 |
