# Supplementary material for: Determinants for cardiovascular disease health check questionnaire: A validation study
Source: PLoS One. 2017 Nov 16;12(11):e0188259. doi: 10.1371/journal.pone.0188259 (PMC5690630; doi:10.1371/journal.pone.0188259)
Supplement: S2 Appendix — (DOCX) [file pone.0188259.s002.docx]

S2 Appendix Questionnaire

- Soalan-soalan di bawah bertujuan untuk mendapat persepsi anda terhadap penyakit kardiovaskular dan pemeriksaan kesihatan untuk pencegahan penyakit kardiovaskular.

**-Penyakit kardiovakular (penyakit CVD)** dalam soalan-soalan berikut adalah merujuk kepada **penyakit serangan jantung dan angin ahmar (strok).**

**-Pemeriksaan kesihatan CVD** dalam soalan-soalan berikut **adalah pemeriksaan untuk mengesan risiko penyakit CVD seperti darah tinggi, kencing manis, masalah kolesterol tinggi, kegemukan, merokok dan kekurangan aktiviti fizikal (senaman).**

- Ianya bukan untuk menguji pengetahuan anda. Oleh itu, **tiada jawapan yang betul atau salah.**
- Sila pilih **satu jawapan** yang paling **sesuai untuk menggambarkan tahap persetujuan anda** terhadap kenyataan tersebut dengan **membulatkan jawapan** anda.

.

***Perception of cardiovascular diseases (e.g. heart disease/stroke) and CVD health checks***

- *The following questionnaire enquires about your perception about various components relating to cardiovascular diseases (CVD) and CVD health check..*

*-Cardiovascular diseases (CVD) in these questions refer to heart attack and stroke.*

*-* CVD *Health check in the following questions refer to health check for CVD prevention which medical examinations whose purpose is to screen for cardiovascular disease risk factors such as high blood pressure, diabetes, high cholesterol, obesity, smoking and lack of physical activity (exercise).*

- *There is no right or wrong answer.*
- *For each statement below, please circle the scale that best describes your level of agreement or disagreement.*

| Skala | Sangat tidak setuju: **STS (1)** | Tidak setuju: **TS (2)** | Tidak pasti :  **TP (3)** | Setuju:  **S (4)** | Sangat setuju: **SS (5)** |
| --- | --- | --- | --- | --- | --- |

| *Scale:* | *Strongly disagree:*  ***SD (1)*** | *Disagree:*  ***D (2)*** | *Not sure :*  ***N (3)*** | *Agree:*  ***A (4)*** | *Strongly agree:*  ***SA (5)*** |
| --- | --- | --- | --- | --- | --- |

| **Bil** | **Soalan** | **Skala Maklumbalas** | | | | |
| --- | --- | --- | --- | --- | --- | --- |
|  |  | **STS**  *SD* | **TS**  *D* | **TP**  *N* | **S**  *A* | **SS**  *SA* |
| A1 | Saya percaya penyakit CVD (contohnya penyakit serangan jantung dan strok) dapat dicegah.  *I believe CVD (for example heart disease, stroke, etc.) can be prevented.* | 1 | 2 | 3 | 4 | 5 |
| A2 | Saya percaya rawatan awal faktor risiko penyakit CVD (contohnya darah tinggi, kolesterol tinggi, kencing manis dan lain-lain) dapat mencegah penyakit CVD.  *I believe early treatment of CVD risk factors (for example high blood pressure, high cholesterol level, diabetes mellitus) can prevent the CVD.* | 1 | 2 | 3 | 4 | 5 |
| A3 | Saya percaya penyakit CVD dapat dirawat.  *I believe CVD is treatable.* | 1 | 2 | 3 | 4 | 5 |
| A4 | Jika penyakit CVD dapat dikesan awal, rawatannya adalah lebih senang.  *If CVD can be detected early, the treatment will be easier.* | 1 | 2 | 3 | 4 | 5 |
| B1 | Saya mempunyai risiko untuk mendapat penyakit CVD.  *I am at risk of CVD.* | 1 | 2 | 3 | 4 | 5 |
| B2 | Umur saya sekarang menjadikan saya berisiko untuk mendapat penyakit CVD.  *My current age puts me at risk of CVD.* | 1 | 2 | 3 | 4 | 5 |
| B3 | Gaya hidup saya menjadikan saya berisiko untuk mendapat penyakit CVD.  *My lifestyle puts me at risk of CVD.* | 1 | 2 | 3 | 4 | 5 |
| B4 | Masalah kesihatan ahli keluarga saya menjadikan saya berisiko untuk mendapat penyakit CVD.  *Medical problems in my family members put me at risk of CVD.* | 1 | 2 | 3 | 4 | 5 |
| B5 | Keadaan kesihatan saya sekarang menjadikan saya berisiko untuk penyakit CVD.  *My current health condition put me at risk of CVD.* | 1 | 2 | 3 | 4 | 5 |

| Skala | Sangat tidak setuju: **STS (1)** | Tidak setuju: **TS (2)** | Tidak pasti :  **TP (3)** | Setuju:  **S (4)** | Sangat setuju: **SS (5)** |
| --- | --- | --- | --- | --- | --- |

| *Scale:* | *Strongly disagree:*  ***SD (1)*** | *Disagree:*  ***D (2)*** | *Neutral :*  ***N (3)*** | *Agree:*  ***A (4)*** | *Strongly agree:*  ***SA (5)*** |
| --- | --- | --- | --- | --- | --- |

| **Bil** | **Soalan** | **Skala Maklumbalas** | | | | |
| --- | --- | --- | --- | --- | --- | --- |
|  |  | **STS**  *SD* | **TS**  *D* | **TP**  *N* | **S**  *A* | **SS**  *SA* |
| Untuk mencegah penyakit kardiovaskular,  *For CVD prevention,* | |  |  |  |  |  |
| C2 | saya lebih suka mengamalkan gaya hidup sihat berbanding menjalani pemeriksaan kesihatan CVD.  *I prefer to adopt a healthy lifestyle than undergoing CVD health check.* | 1 | 2 | 3 | 4 | 5 |
| C3 | saya lebih yakin mengamalkan gaya hidup sihat berbanding menggunakan rawatan perubatan.  *I am more confident with practising healthy lifestyle than using medical treatment.* | 1 | 2 | 3 | 4 | 5 |

| Skala | Sangat tidak setuju: **STS (1)** | Tidak setuju: **TS (2)** | Tidak pasti :  **TP (3)** | Setuju:  **S (4)** | Sangat setuju: **SS (5)** |
| --- | --- | --- | --- | --- | --- |

| *Scale:* | *Strongly disagree:*  ***SD (1)*** | *Disagree:*  ***D (2)*** | *Neutral :*  ***N (3)*** | *Agree:*  ***A (4)*** | *Strongly agree:*  ***SA (5)*** |
| --- | --- | --- | --- | --- | --- |

| **Bil** | **Soalan** | **Skala Maklumbalas** | | | | |
| --- | --- | --- | --- | --- | --- | --- |
|  |  | **STS**  *SD* | **TS**  *D* | **TP**  *N* | **S**  *A* | **SS**  *SA* |
| DB1 | Saya rasa menjalani pemeriksaan kesihatan CVD dapat memberi jaminan kesihatan saya.  *I feel undergoing CVD health check will give assurance for my health.* | 1 | 2 | 3 | 4 | 5 |
| DB2 | Kita tidak akan tahu tahap kesihatan CVD diri jika tidak menjalani pemeriksaan kesihatan CVD.  *We will not know our CVD health status if we do not undergo CVD health checks.* | 1 | 2 | 3 | 4 | 5 |
| DB3 | Pemeriksaan kesihatan CVD akan memberi petunjuk untuk pencegahan penyakit CVD.  *CVD health check can act as an indicator for CVD prevention.* | 1 | 2 | 3 | 4 | 5 |
| DB4 | Pemeriksaan kesihatan CVD membolehkan kita mengesan awal faktor risiko penyakit jantung/strok (contoh darah tinggi, kolesterol tinggi, kencing manis dan lain-lain).  *CVD health check enables us to detect risk factors of heart disease/stroke early.* | 1 | 2 | 3 | 4 | 5 |

| Skala | Sangat tidak setuju: **STS (1)** | Tidak setuju: **TS (2)** | Tidak pasti :  **TP (3)** | Setuju:  **S (4)** | Sangat setuju: **SS (5)** |
| --- | --- | --- | --- | --- | --- |

| *Scale:* | *Strongly disagree:*  ***SD (1)*** | *Disagree:*  ***D (2)*** | *Neutral :*  ***N (3)*** | *Agree:*  ***A (4)*** | *Strongly agree:*  ***SA (5)*** |
| --- | --- | --- | --- | --- | --- |

| **Bil** | **Soalan** | **Skala Maklumbalas** | | | | |
| --- | --- | --- | --- | --- | --- | --- |
|  |  | **STS**  *SD* | **TS**  *D* | **TP**  *N* | **S**  *A* | **SS**  *SA* |
| Pemeriksaan CVD  *CVD health check* | |  |  |  |  |  |
| DD1 | adalah pembaziran masa.  *is a waste of time.* | 1 | 2 | 3 | 4 | 5 |
| DD2 | adalah pembaziran wang.  *is a waste of money.* | 1 | 2 | 3 | 4 | 5 |
| DD3 | melibatkan prosedur yang menyusahkan. (contohnya: keperluaan berpuasa sebelum pemeriksaan darah).  *involves a troublesome procedure. (e.g. the need to fast before blood test.)* | 1 | 2 | 3 | 4 | 5 |
| DD4 | yang mendapati keputusan tidak normal akan menimbulkan masalah. (contohnya menjejaskan peluang untuk membeli insurans atau mendapatkan kerja).  *which found abnormal health check results will give rise to problems. (e.g. affect the chance to purchase insurance or get a job).* | 1 | 2 | 3 | 4 | 5 |

| Skala | Sangat tidak setuju: **STS (1)** | Tidak setuju: **TS (2)** | Tidak pasti :  **TP (3)** | Setuju:  **S (4)** | Sangat setuju: **SS (5)** |
| --- | --- | --- | --- | --- | --- |

| *Scale:* | *Strongly disagree:*  ***SD (1)*** | *Disagree:*  ***D (2)*** | *Neutral :*  ***N (3)*** | *Agree:*  ***A (4)*** | *Strongly agree:*  ***SA (5)*** |
| --- | --- | --- | --- | --- | --- |

| **Bil** | **Soalan** | **Skala Maklumbalas** | | | | |
| --- | --- | --- | --- | --- | --- | --- |
|  |  | **STS**  *SD* | **TS**  *D* | **TP**  *N* | **S**  *A* | **SS**  *SA* |
| RFR1 | Saya bersedia untuk menghadapi keputusan pemeriksaan kesihatan CVD.  *I am ready to face the results of the CVD health check.* | 1 | 2 | 3 | 4 | 5 |
| RFR2 | Saya ingin mendapat tahu tahap kesihatan CVD saya.  *I want to know my CVD health status.* | 1 | 2 | 3 | 4 | 5 |
| RFR3 | Saya tidak mahu berfikir dan tidak ingin mengambil tahu langsung mengenai penyakit CVD.  *I don’t want to think and know about CVD diseases at all.* | 1 | 2 | 3 | 4 | 5 |

| **Bil** | **Soalan** | **Skala Maklumbalas** | | | | |
| --- | --- | --- | --- | --- | --- | --- |
|  |  | **STS**  *SD* | **TS**  *D* | **TP**  *N* | **S**  *A* | **SS**  *SA* |
| Jika keputusan pemeriksaan kesihatan CVD tidak normal,  *If the CVD health check results are abnormal,* | |  |  |  |  |  |
| RHO1 | saya bersedia untuk mengambil rawatan ubat.  *I am ready to take medication.* | 1 | 2 | 3 | 4 | 5 |
| RHO2 | saya bersedia untuk mengubah gaya hidup saya.  *I am ready to adjust my lifestyle.* | 1 | 2 | 3 | 4 | 5 |
| RHO3 | saya bersedia untuk menanggung kos rawatan berikutnya.  *I am ready to bear the cost of subsequent treatment.* | 1 | 2 | 3 | 4 | 5 |
| RHO4 | Saya tidak bersedia untuk melakukan apa-apa.  *I am not ready to do anything.* |  |  |  |  |  |

| Skala | Sangat tidak setuju: **STS (1)** | Tidak setuju: **TS (2)** | Tidak pasti :  **TP (3)** | Setuju:  **S (4)** | Sangat setuju: **SS (5)** |
| --- | --- | --- | --- | --- | --- |

| *Scale:* | *Strongly disagree:*  ***SD (1)*** | *Disagree:*  ***D (2)*** | *Neutral :*  ***N (3)*** | *Agree:*  ***A (4)*** | *Strongly agree:*  ***SA (5)*** |
| --- | --- | --- | --- | --- | --- |

| **Bil** | **Soalan** | **Skala Maklumbalas** | | | | |
| --- | --- | --- | --- | --- | --- | --- |
|  |  | **STS**  *SD* | **TS**  *D* | **TP**  *N* | **S**  *A* | **SS**  *SA* |
| F1 | Saya akan berusaha untuk meluangkan masa untuk menjalani pemeriksaan kesihatan CVD.  *I will make the effort to allocate time to go for a CVD health check.* | 1 | 2 | 3 | 4 | 5 |
| F2 | Bagi saya, kos pemeriksaan kesihatan CVD adalah membebankan.  *The cost of doing a CVD health checks is a burden for me.* | 1 | 2 | 3 | 4 | 5 |
| F3 | Tempat pemeriksaan kesihatan CVD adalah jauh dari rumah/tempat kerja saya.  *The place for CVD health checks is far from my house/workplace.* | 1 | 2 | 3 | 4 | 5 |
| F4 | Saya ada masalah pengangkutan untuk pergi menjalani pemeriksaan kesihatan CVD.  *I have a problem with transportation to go for CVD health check .* | 1 | 2 | 3 | 4 | 5 |

| Skala | Sangat tidak setuju: **STS (1)** | Tidak setuju: **TS (2)** | Tidak pasti :  **TP (3)** | Setuju:  **S (4)** | Sangat setuju: **SS (5)** |
| --- | --- | --- | --- | --- | --- |

| *Scale:* | *Strongly disagree:*  ***SD (1)*** | *Disagree:*  ***D (2)*** | *Neutral :*  ***N (3)*** | *Agree:*  ***A (4)*** | *Strongly agree:*  ***SA (5)*** |
| --- | --- | --- | --- | --- | --- |

| **Bil** | **Soalan** | **Skala Maklumbalas** | | | | |
| --- | --- | --- | --- | --- | --- | --- |
|  |  | **STS**  *SD* | **TS**  *D* | **TP**  *N* | **S**  *A* | **SS**  *SA* |
| Saya akan menjalanin pemeriksaan kesihatan CVD  *I will perform the CVD health check* | |  |  |  |  |  |
| G1 | jika dinasihatkan oleh doktor.  *if recommended to do so by doctors.* | 1 | 2 | 3 | 4 | 5 |
| G2 | jika dinasihatkan oleh ahli keluarga saya.  *if my family member advises me to do so.* | 1 | 2 | 3 | 4 | 5 |
| G3 | jika dinasihatkan oleh kawan saya.  *if my friend advises me to do so.* | 1 | 2 | 3 | 4 | 5 |
| G4 | jika majikan memerlukan saya berbuat demikian.  *if my employer requires me to do so.* | 1 | 2 | 3 | 4 | 5 |
| G5 | kerana orang-orang sekeliling saya telah berbuat demikian.  *as people around me have already done so.* | 1 | 2 | 3 | 4 | 5 |

**Keinginan untuk menjalani pemeriksaan kesihatan CVD**

**Kenyataan berikut berkenaan dengan keinginan/niat anda untuk menjalani pemeriksaan kesihatan untuk pencegahan penyakit kardiovaskular.**

Sila pilih **satu jawapan** yang paling sesuai untuk menggambarkan tahap persetujuan anda terhadap kenyataan tersebut dengan **membulatkan jawapan** anda.

***The intention to undergo CVD health checks.***

*The following statement is about your intention to participate in the health check for CVD prevention.*

*For each statement below, please circle the scale that best describes your level of intention.*

| Skala  *Scale* | Sangat tidak mungin:  **STM (1)** | Tidak mungkin:  **TM (2)** | Tidak pasti :  **TP (3)** | Mungkin :  **M (4)** | Sangat mungkin:  **SM (5)** |
| --- | --- | --- | --- | --- | --- |

| Scale | Very unlikely:  **VUL (1)** | Unlikely:  **UL (2)** | Not sure :  **NS (3)** | Likely:  **L (4)** | Very likely:  **VL (5)** |
| --- | --- | --- | --- | --- | --- |

| **Bil**  **no** | **Soalan**  **Item** | **Skala Maklumbalas**  **Response Scale** | | | | | |
| --- | --- | --- | --- | --- | --- | --- | --- |
|  |  | **STM**  ***VUL*** | **TM*UL*** | **TP**  ***NS*** | | **M**  ***L*** | **SM**  ***VL*** |
| 1 | Apa kemungkinan anda untuk pergi menjalani pemeriksaan kesihatan CVD dalam masa berikut:  *How likely are you going to undergo CVD health checks within the following time period:* |  |  |  |  | |  |
|  | 1. Dalam 3 bulan yang akan datang?   *Within the next 3 months?* | 1 | 2 | 3 | 4 | | 5 |
|  | 1. Dalam 6 bulan yang akan datang?   *Within the next 6 months?* | 1 | 2 | 3 | 4 | | 5 |
|  | 1. Dalam tempoh 1 tahun yang akan datang?   *Within the next 1 year?* | 1 | 2 | 3 | 4 | | 5 |
