# Supplementary material for: Determinants for cardiovascular disease health check questionnaire: A validation study
Source: PLoS One. 2017 Nov 16;12(11):e0188259. doi: 10.1371/journal.pone.0188259 (PMC5690630; doi:10.1371/journal.pone.0188259)
Supplement: S3 Appendix — (DOCX) [file pone.0188259.s003.docx]

**S3 Appendix : The mean, standard deviation of items and correlation matrix of each concept**

The mean, standard deviation of items and correlation matrix of each concept are shown in Tables 1 to 9, respectively.

| **Table 1: Mean, standard deviation of items and correlation matrix for concept of “Believe that the disease course can be changed for better outcomes”** |
| --- |
| \|  \| Mean score \| Standard  deviation \| A1 \| A2 \| A3 \| A4 \| \| --- \| --- \| --- \| --- \| --- \| --- \| --- \| \| A1 \| 4.13 \| 0.74 \| 1.000 \| 0.547 \| 0.467 \| 0.412 \| \| A2 \| 4.27 \| 0.63 \|  \| 1.000 \| 0.528 \| 0.512 \| \| A3 \| 4.04 \| 0.72 \|  \|  \| 1.000 \| 0.520 \| \| A4 \| 4.41 \| 0.57 \|  \|  \|  \| 1.000 \| |

| Table 2: Mean, standard deviation of items and correlation matrix for concept of “Perceived self at risk of CVD” |
| --- |
| \|  \| Mean score \| Standard  deviation \| B1 \| B2 \| B3 \| B4 \| B5 \| \| --- \| --- \| --- \| --- \| --- \| --- \| --- \| --- \| \| B1 \| 3.34 \| 0.90 \| 1.000 \| 0.644 \| 0.485 \| 0.456 \| 0.573 \| \| B2 \| 3.54 \| 0.88 \|  \| 1.000 \| 0.444 \| 0.383 \| 0.433 \| \| B3 \| 3.16 \| 0.96 \|  \|  \| 1.000 \| 0.304 \| 0.541 \| \| B4 \| 3.05 \| 1.01 \|  \|  \|  \| 1.000 \| 0.503 \| \| B5 \| 2.89 \| 1.00 \|  \|  \|  \|  \| 1.000 \| |

| Table 3: Mean, standard deviation of items and correlation matrix for concept of “Preferred method for CVD prevention” |
| --- |
| \|  \| Mean score \| Standard  deviation \| C1 \| C2 \| C3 \| \| --- \| --- \| --- \| --- \| --- \| --- \| \| C1 \| 4.06 \| 0.87 \| 1.000 \| 0.333 \| 0.242 \| \| C2 \| 3.53 \| 1.03 \|  \| 1.000 \| 0.604 \| \| C3 \| 3.74 \| 0.98 \|  \|  \| 1.000 \| |
| Table 4: Mean, standard deviation of items and correlation matrix for concept of “Perceived benefits of health checks” |
| \|  \| Mean score \| Standard  deviation \| DB1 \| DB2 \| DB3 \| DB4 \| \| --- \| --- \| --- \| --- \| --- \| --- \| --- \| \| DB1 \| 3.94 \| 0.79 \| 1.000 \| 0.484 \| 0.493 \| 0.417 \| \| DB2 \| 4.23 \| 0.59 \|  \| 1.000 \| 0.682 \| 0.628 \| \| DB3 \| 4.23 \| 0.54 \|  \|  \| 1.000 \| 0.716 \| \| DB4 \| 4.37 \| 0.52 \|  \|  \|  \| 1.000 \| |

| Table 5: Mean, standard deviation of items and correlation matrix for concept of “Perceived drawbacks of health checks” |
| --- |
| \|  \| Mean score \| Standard  deviation \| DD1 \| DD2 \| DD3 \| DD4 \| \| --- \| --- \| --- \| --- \| --- \| --- \| --- \| \| DD1 \| 1.78 \| 0.67 \| 1.000 \| 0.838 \| 0.577 \| 0.380 \| \| DD2 \| 1.81 \| 0.68 \|  \| 1.000 \| 0.563 \| 0.344 \| \| DD3 \| 2.00 \| 0.82 \|  \|  \| 1.000 \| 0.385 \| \| DD4 \| 2.71 \| 1.08 \|  \|  \|  \| 1.000 \| |
| Table 6: Mean, standard deviation of items and correlation matrix for concept of “Readiness to know the result of health checks” |
| \|  \| Mean score \| Standard  deviation \| RFR1 \| RFR2 \| RFR3* \| \| --- \| --- \| --- \| --- \| --- \| --- \| \| RFR1 \| 4.07 \| 0.53 \| 1.000 \| 0.676 \| 0.380 \| \| RFR2 \| 4.17 \| 0.54 \|  \| 1.000 \| 0.511 \| \| RFR3* \| 4.09 \| 0.76 \|  \|  \| 1.000 \| \| *reverse scoring \| \| \| \| \| \| |

| Table 7: Mean, standard deviation of items and correlation matrix for concept of “Readiness to handle the outcomes following health checks” |
| --- |
| \|  \| Mean score \| Standard  deviation \| RHO1 \| RHO2 \| RHO3 \| RHO4* \| \| --- \| --- \| --- \| --- \| --- \| --- \| --- \| \| RHO1 \| 3.88 \| 0.75 \| 1.000 \| 0.336 \| 0.526 \| 0.298 \| \| RHO2 \| 4.19 \| 0.55 \|  \| 1.000 \| 0.342 \| 0.542 \| \| RHO3 \| 3.73 \| 0.78 \|  \|  \| 1.000 \| 0.351 \| \| RHO4* \| 4.02 \| 0.74 \|  \|  \|  \| 1.000 \| \| *reverse scoring \| \| \| \| \| \| \| |

| Table 8: Mean, standard deviation of items and correlation matrix for concept of “External barriers” |
| --- |
| \|  \| Mean score \| Standard  deviation \| F1* \| F2 \| F3 \| F4 \| \| --- \| --- \| --- \| --- \| --- \| --- \| --- \| \| F1* \| 1.95 \| 0.54 \| 1.000 \| 0.304 \| 0.292 \| 0.263 \| \| F2 \| 2.65 \| 0.95 \|  \| 1.000 \| 0.396 \| 0.241 \| \| F3 \| 2.45 \| 0.87 \|  \|  \| 1.000 \| 0.496 \| \| F4 \| 2.14 \| 0.82 \|  \|  \|  \| 1.000 \| \| *reverse scoring \| \| \| \| \| \| \| |
| Table 9: Mean, standard deviation of items and correlation matrix for concept of “Influence by significant others” |
| \|  \| Mean \| Standard  deviation \| G1 \| G2 \| G3 \| G4 \| G5 \| \| --- \| --- \| --- \| --- \| --- \| --- \| --- \| --- \| \| G1 \| 4.18 \| 0.60 \| 1.000 \| 0.622 \| 0.494 \| 0.583 \| 0.361 \| \| G2 \| 3.9 \| 0.75 \|  \| 1.000 \| 0.749 \| 0.577 \| 0.498 \| \| G3 \| 3.73 \| 0.82 \|  \|  \| 1.000 \| 0.554 \| 0.560 \| \| G4 \| 4.06 \| 0.65 \|  \|  \|  \| 1.000 \| 0.411 \| \| G5 \| 3.51 \| 0.94 \|  \|  \|  \|  \| 1.000 \| |
