# Supplementary material for: Determinants for cardiovascular disease health check questionnaire: A validation study
Source: PLoS One. 2017 Nov 16;12(11):e0188259. doi: 10.1371/journal.pone.0188259 (PMC5690630; doi:10.1371/journal.pone.0188259)
Supplement: S4 Appendix — (DOCX) [file pone.0188259.s004.docx]

| **S4 Appendix: Summary of test-retest reliability for all items in the questionnaire**   \| Item \| Unweighted kappa  (Strength of agreement) \| \| \| Weighted kappa with linear weighting  (Strength of agreement) \| \| Weighted kappa with quadratic weighting  (Strength of agreement) \| \| \| --- \| --- \| --- \| --- \| --- \| --- \| --- \| --- \| \| A1 \| 0.334 \| \| (fair) \| 0.364 \| (fair) \| 0.417 \| (moderate) \| \| A2 \| 0.433 \| \| (moderate) \| 0.587 \| (moderate) \| 0.576 \| (moderate) \| \| A3 \| 0.353 \| \| (fair) \| 0.423 \| (moderate) \| 0.516 \| (moderate) \| \| A4 \| 0.327 \| \| (fair) \| 0.324 \| (fair) \| 0.319 \| (fair) \| \|  \|  \| \|  \|  \|  \|  \|  \| \| B1 \| 0.418 \| \| (moderate) \| 0.491 \| (moderate) \| 0.584 \| (moderate) \| \| B2 \| 0.198 \| \| (slight) \| 0.268 \| (fair) \| 0.367 \| (fair) \| \| B3 \| 0.283 \| \| (fair) \| 0.390 \| (fair) \| 0.519 \| (moderate) \| \| B4 \| 0.369 \| \| (fair) \| 0.474 \| (moderate) \| 0.576 \| (moderate) \| \| B5 \| 0.219 \| \| (fair) \| 0.352 \| (fair) \| 0.479 \| (moderate) \| \|  \|  \| \|  \|  \|  \|  \|  \| \| C2 \| 0.329 \| \| (fair) \| 0.382 \| (fair) \| 0.404 \| (moderate) \| \| C3 \| 0.333 \| \| (fair) \| 0.470 \| (moderate) \| 0.615 \| (substantial) \| \|  \|  \| \|  \|  \|  \|  \|  \| \| DB1 \| 0.328 \| \| (fair) \| 0.339 \| (fair) \| 0.360 \| (fair) \| \| DB2 \| 0.214 \| \| (fair) \| 0.272 \| (fair) \| 0.370 \| (fair) \| \| DB3 \| 0.277 \| \| (fair) \| 0.307 \| (fair) \| 0.361 \| (fair) \| \| DB4 \| 0.381 \| \| (fair) \| 0.409 \| (moderate) \| 0.460 \| (moderate) \| \|  \|  \| \|  \|  \|  \|  \|  \| \| DD1 \| 0.255 \| (fair) \| \| 0.365 \| (fair) \| 0.510 \| (moderate) \| \| DD2 \| 0.179 \| (slight) \| \| 0.331 \| (fair) \| 0.518 \| (moderate) \| \| DD3 \| 0.279 \| (fair) \| \| 0.351 \| (fair) \| 0.450 \| (moderate) \| \| DD4 \| 0.229 \| (fair) \| \| 0.259 \| (fair) \| 0.302 \| (fair) \| \|  \|  \|  \| \|  \|  \|  \|  \| \| RFR1 \| 0.320 \| (fair) \| \| 0.357 \| (fair) \| 0.418 \| (moderate) \| \| RFR2 \| 0.361 \| (fair) \| \| 0.393 \| (fair) \| 0.448 \| (moderate) \| \| RFR3 \| 0.284 \| (fair) \| \| 0.336 \| (fair) \| 0.397 \| (fair) \| \|  \|  \|  \| \|  \|  \|  \|  \| \| RHO1 \| 0.361 \| (fair) \| \| 0.511 \| (moderate) \| 0.688 \| (substantial) \| \| RHO2 \| 0.409 \| (moderate) \| \| 0.413 \| (moderate) \| 0.426 \| (moderate) \| \| RHO3 \| 0.513 \| (moderate) \| \| 0.533 \| (moderate) \| 0.561 \| (moderate) \| \| RHO4 \| 0.280 \| (fair) \| \| 0.341 \| (fair) \| 0.428 \| (moderate) \| \|  \|  \|  \| \|  \|  \|  \|  \| \| F1 \| 0.355 \| (fair) \| \| 0.374 \| (fair) \| 0.405 \| (moderate) \| \| F2 \| 0.319 \| (fair) \| \| 0.372 \| (fair) \| 0.414 \| (moderate) \| \| F3 \| 0.462 \| (moderate) \| \| 0.495 \| (moderate) \| 0.496 \| (moderate) \| \| F4 \| 0.350 \| (fair) \| \| 0.513 \| (moderate) \| 0.682 \| (substantial) \| \|  \|  \|  \| \|  \|  \|  \|  \| \| G1 \| 0.346 \| (fair) \| \| 0.322 \| (fair) \| 0.281 \| (fair) \| \| G2 \| 0.340 \| (fair) \| \| 0.440 \| (moderate) \| 0.547 \| (moderate) \| \| G3 \| 0.189 \| (slight) \| \| 0.264 \| (fair) \| 0.354 \| (fair) \| \| G4 \| 0.344 \| (fair) \| \| 0.377 \| (fair) \| 0.403 \| (moderate) \| \| G5 \| 0.218 \| (fair) \| \| 0.354 \| (fair) \| 0.493 \| (moderate) \| \|  \|  \|  \| \|  \|  \|  \|  \| |
| --- | --- | --- | --- | --- | --- | --- | --- | --- | --- | --- | --- | --- | --- | --- | --- | --- | --- | --- | --- | --- | --- | --- | --- | --- | --- | --- | --- | --- | --- | --- | --- | --- | --- | --- | --- | --- | --- | --- | --- | --- | --- | --- | --- | --- | --- | --- | --- | --- | --- | --- | --- | --- | --- | --- | --- | --- | --- | --- | --- | --- | --- | --- | --- | --- | --- | --- | --- | --- | --- | --- | --- | --- | --- | --- | --- | --- | --- | --- | --- | --- | --- | --- | --- | --- | --- | --- | --- | --- | --- | --- | --- | --- | --- | --- | --- | --- | --- | --- | --- | --- | --- | --- | --- | --- | --- | --- | --- | --- | --- | --- | --- | --- | --- | --- | --- | --- | --- | --- | --- | --- | --- | --- | --- | --- | --- | --- | --- | --- | --- | --- | --- | --- | --- | --- | --- | --- | --- | --- | --- | --- | --- | --- | --- | --- | --- | --- | --- | --- | --- | --- | --- | --- | --- | --- | --- | --- | --- | --- | --- | --- | --- | --- | --- | --- | --- | --- | --- | --- | --- | --- | --- | --- | --- | --- | --- | --- | --- | --- | --- | --- | --- | --- | --- | --- | --- | --- | --- | --- | --- | --- | --- | --- | --- | --- | --- | --- | --- | --- | --- | --- | --- | --- | --- | --- | --- | --- | --- | --- | --- | --- | --- | --- | --- | --- | --- | --- | --- | --- | --- | --- | --- | --- | --- | --- | --- | --- | --- | --- | --- | --- | --- | --- | --- | --- | --- | --- | --- | --- | --- | --- | --- | --- | --- | --- | --- | --- | --- | --- | --- | --- | --- | --- | --- | --- | --- | --- | --- | --- | --- | --- | --- | --- | --- | --- | --- | --- | --- | --- | --- | --- | --- | --- | --- | --- | --- | --- | --- | --- | --- | --- | --- | --- | --- | --- | --- | --- | --- | --- | --- | --- | --- | --- | --- | --- | --- | --- | --- | --- | --- | --- | --- | --- | --- | --- | --- | --- | --- | --- | --- | --- | --- | --- | --- | --- | --- | --- | --- | --- | --- | --- | --- | --- | --- | --- | --- | --- | --- | --- | --- | --- | --- | --- | --- | --- | --- | --- | --- | --- | --- | --- | --- | --- | --- | --- | --- | --- | --- | --- | --- | --- | --- | --- | --- | --- | --- | --- | --- | --- | --- | --- |
